# Supplementary material for: An essential role of acetyl coenzyme A in the catalytic cycle of insect arylalkylamine N-acetyltransferase
Source: Commun Biol. 2020 Aug 14;3:441. doi: 10.1038/s42003-020-01177-9 (PMC7427786; doi:10.1038/s42003-020-01177-9)
Supplement: Supplementary file 1 — Supplementary Information [file 42003_2020_1177_MOESM1_ESM.pdf]

## Supplementary Figures

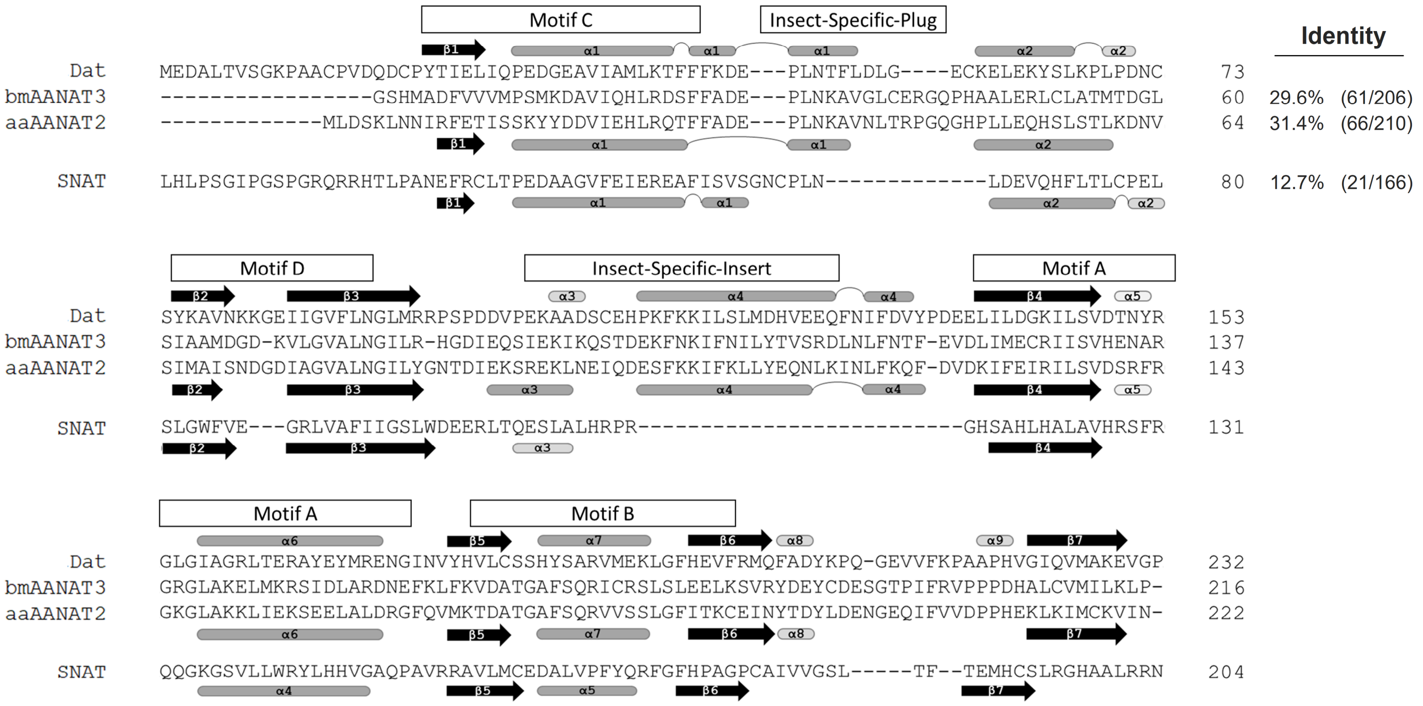


**Supplementary Figure 1. Multiple sequence alignment among AANATs with secondary structural information.**

Multiple sequence alignment with secondary structural information among SNAT (from Oris aries) and insect AANATs, including Dat (from Drosophila melanogaster), bmAANAT3 (from Bombyx mori), and aaAANAT2 (from Aedes aegypti). The secondary structural information was according to the crystal structures, SNAT (SNAT/CoA-S-Acetyl-Tryptamine; PDB code: 1CJW), Dat (Dat/CoA/Ac-TRYP complex, PDB code: 5GI9), and aaAANAT2 (apo form, PDB code: 4FD6). Sequence identities are shown in right. Parentheses = number of identical residues in the aligned regions/inquiry protein sequence length.

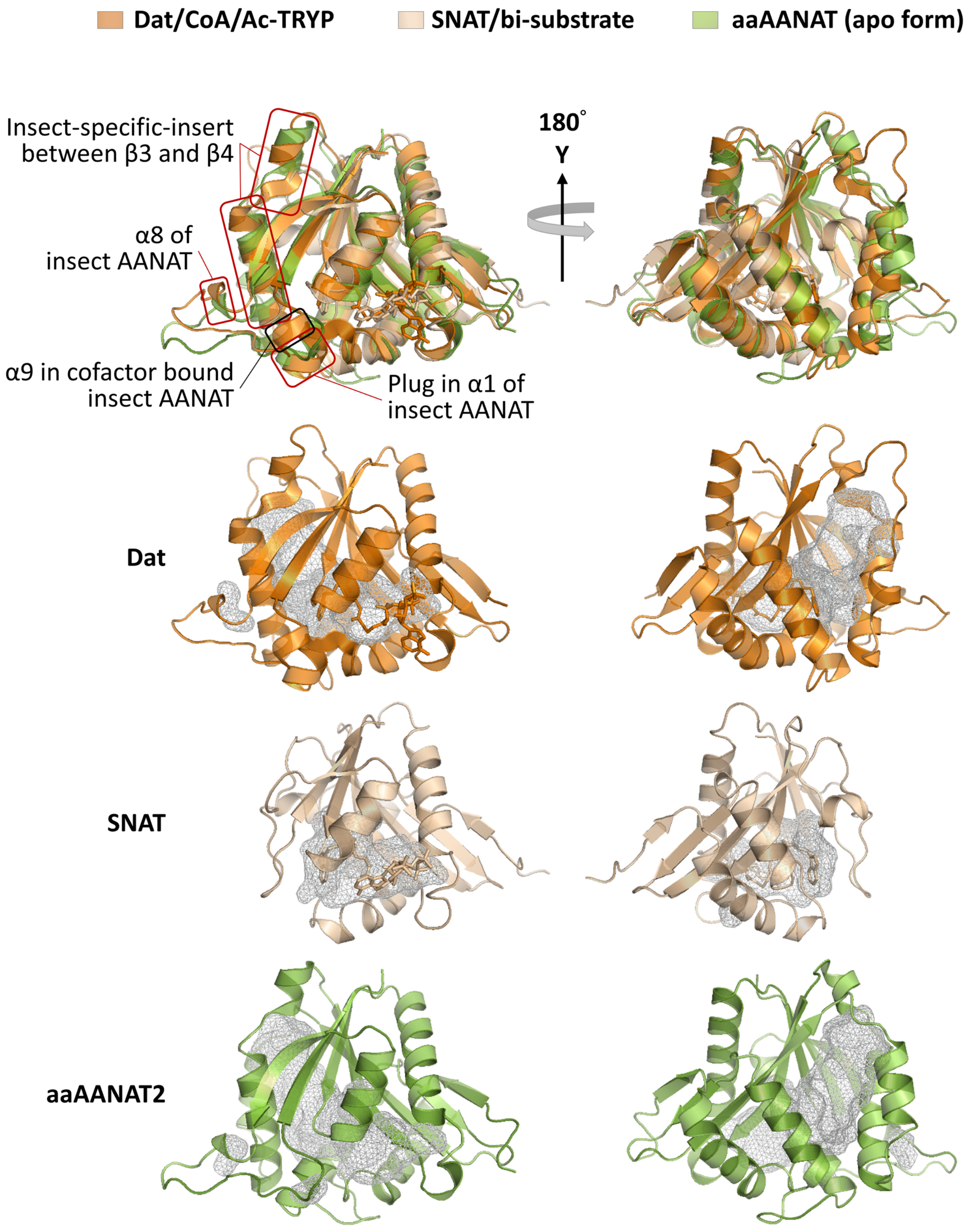


**Supplementary Figure 2. Comparison of AANATs with known structures.**

Comparison of overall structures and cavities of Dat (from *Drosophila melanogaster*), aaAANAT2 (from *Aedes aegypti*), and SNAT (from *Oris aries*). Dat (Dat/CoA/Ac-TRYP complex, PDB code: 5GI9) is orange colored, aaAANAT2 (apo form, PDB code: 4FD6) is green colored, and SNAT (SNAT/CoA-acetyl-serotonin; PDB code: 1CJW) is wheat colored. The solvent-accessible cavities are shown in wireframe.

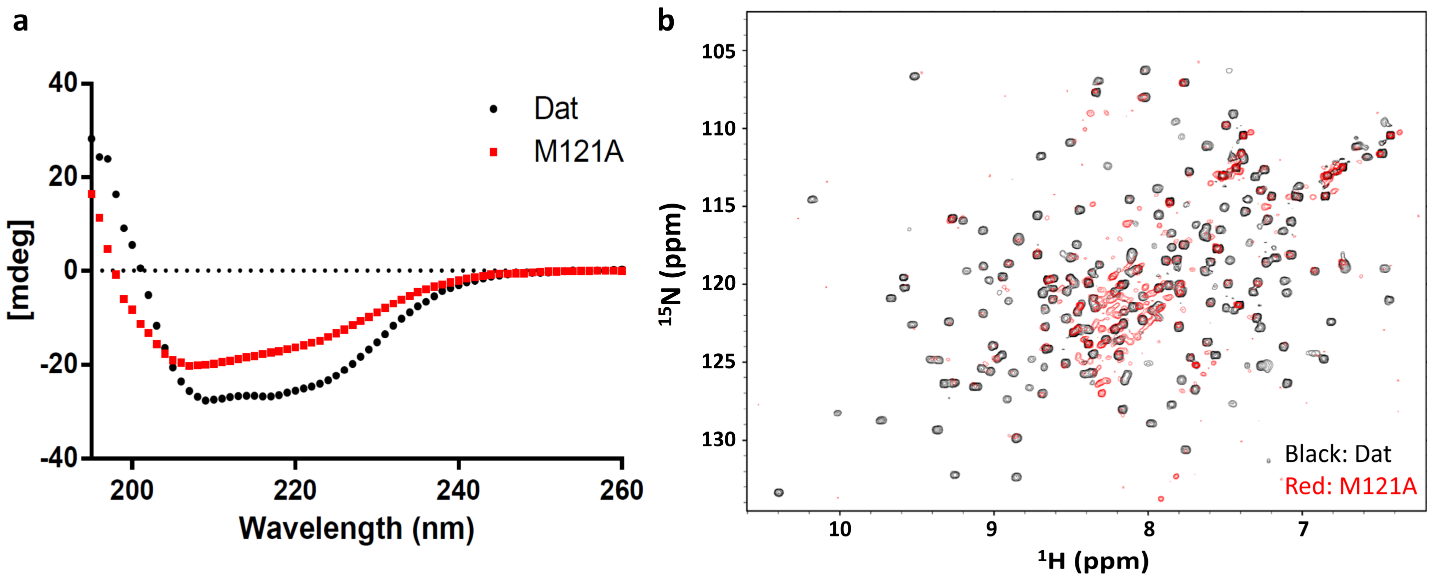


**Supplementary Figure 3. Comparison of secondary structures and tertiary structures of Dat and M121A.**

**a,** Far-UV CD spectra of Dat (black) and M121A mutant (red). **b,** 2D $^{1}$H-$^{15}$N HSQC spectra of Dat (black) and M121A mutant (red).

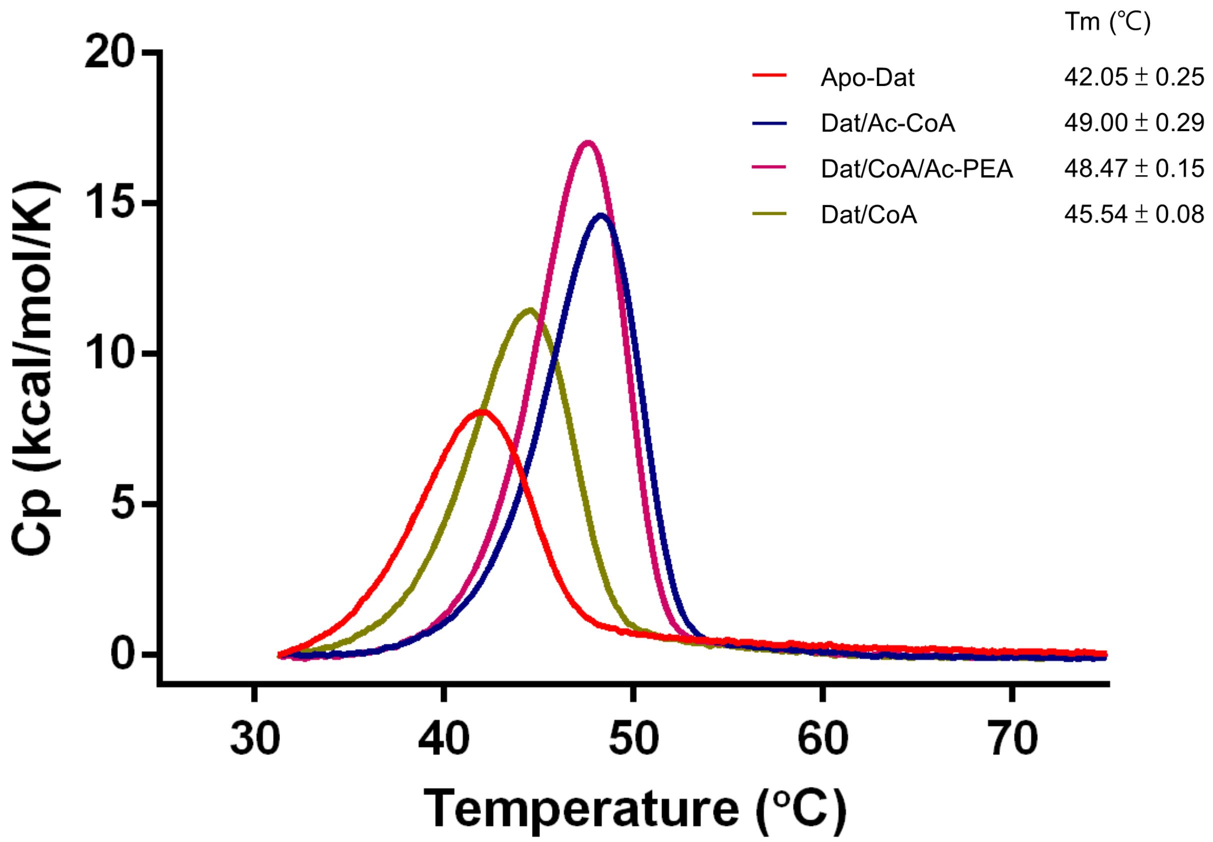


**Supplementary Figure 4. DSC data showed thermostability of Dat in different forms.**

Data for the apo-Dat, Dat/Ac-CoA binary complex, Dat/CoA/Ac-PEA ternary complex and Dat/CoA binary complex are shown in red, blue, magenta and green, respectively. Tm values were calculated as mean ± sd with n = 3 biologically independent experiments.

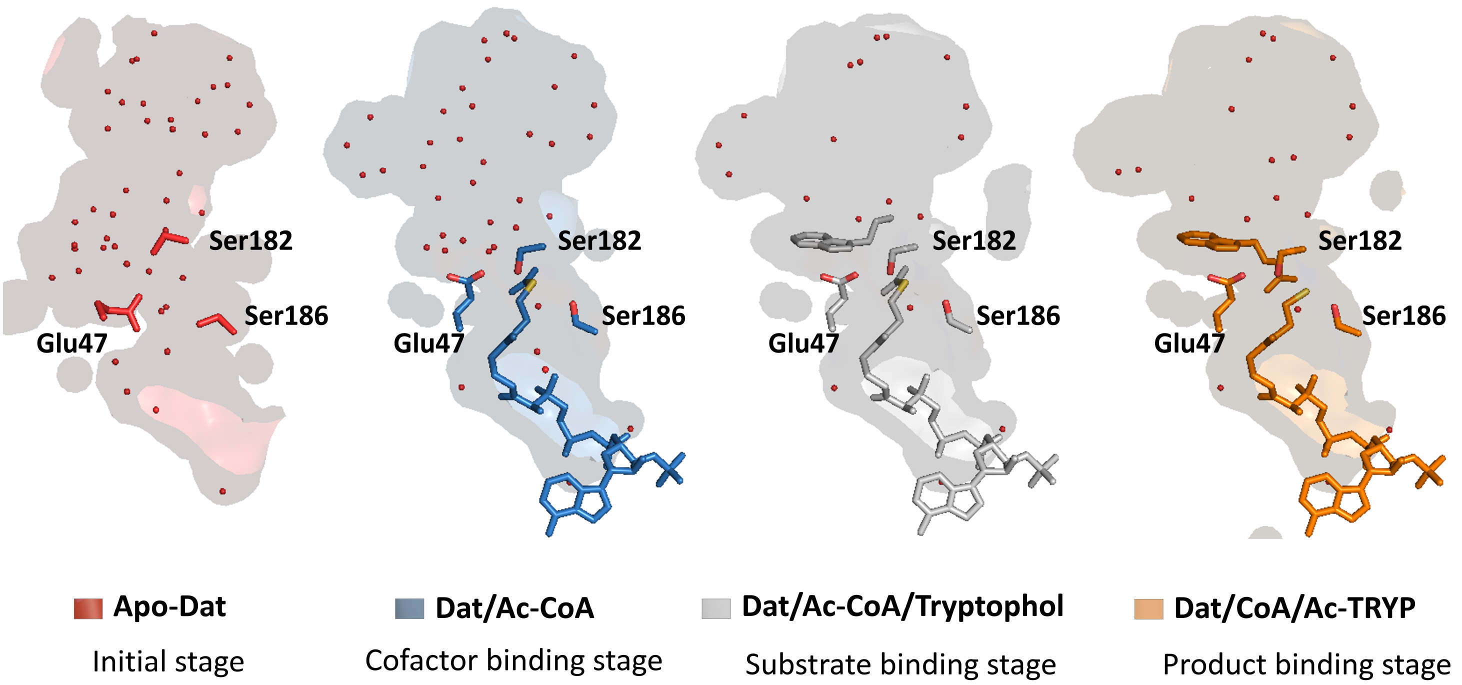


**Supplementary Figure 5. Water molecules in the reaction tunnel of Dat.**

Water molecules in the Dat reaction tunnel are shown in different stages of Dat. Crystal structure of Dat in the initial stage (apo-Dat, PDB code: 3V8I), the cofactor binding stage (Dat/Ac-CoA, PDB code: 3TE4), the substrate binding stage (Dat/Ac-CoA/tryptophol, PDB code: 6K80), and the product binding stage (Dat/CoA/Ac-TRYP, PDB code: 5GI9) are colored red, blue, gray, and orange, respectively. Red spheres represent water molecules. Catalytic residues and ligands are shown as sticks.

**Supplementary Tables**

**Supplementary Table 1. Detailed protein-reactant contacts of Dat/CoA/Ac-TRYP**

The number represents the distance (Å) between ligand atoms and protein residue atoms. Detail of ligands with labeled atoms are shown in the right panel.

(i) Ac-TRYP hydrogen bonds

| Ac-TRYP Atom | Protein Residue Atom | Distance (Å) |
|---|---|---|
| O | Leu$^{146}$ N | 2.9 |
| N | Leu$^{180}$ O | 3.0 |

(ii) Ac-TRYP van der Waals interactions

| Ac-TRYP Atom | Protein Residue/CoA Atom | Distance (Å) | Ac-TRYP Atom | Protein Residue/CoA Atom | Distance (Å) |
|---|---|---|---|---|---|
| C01 | Phe$^{43}$ CZ | 3.9 | C09 | Leu$^{61}$ CD1 | 3.5 |
| | Met$^{121}$ CE | 3.9 | | Tyr$^{64}$ CE1 | 3.9 |
| C03 | Phe$^{43}$ CZ | 3.7 | | Ile$^{145}$ CD1 | 3.7 |
| | Met$^{121}$ CE | 3.7 | | Phe$^{114}$ CE1 | 3.8 |
| C04 | Phe$^{43}$ CZ | 3.8 | C10 | Leu$^{61}$ CD1 | 3.5 |
| | Ile$^{117}$ CG2 | 3.8 | | Phe$^{114}$ CE1 | 3.4 |
| C05 | CoA C2P | 3.6 | C11 | CoA C2P | 3.5 |
| C06 | Glu$^{47}$ CG | 3.7 | | Lys$^{144}$ CO | 3.8 |
| | Ser$^{182}$ CB | 3.9 | C | Lys$^{144}$ CO | 3.8 |
| C07 | Met$^{121}$ CE | 3.4 | | Val$^{179}$ CG1 | 3.8 |
| | Ile$^{145}$ CD1 | 3.5 | | CoA S1P | 3.8 |
| C08 | Ile$^{117}$ CD1 | 3.7 | | | |

(iii) CoA salt bridge

| CoA Atom | Protein Residue Atom | Distance (Å) |
|---|---|---|
| O2A | Lys$^{192}$ NZ | 2.7 |
| O9A | Lys$^{192}$ NZ | 2.7 |

(iv) CoA hydrogen bonds

| CoA Atom | Protein Residue Atom | Distance (Å) | CoA Atom | Protein Residue Atom | Distance (Å) |
|---|---|---|---|---|---|
| N4P | Leu$^{146}$ O | 2.8 | O4A | Ala$^{158}$ N | 2.9 |
| O9P | Val$^{148}$ N | 3.1 | O5A | Gly$^{154}$ N | 2.8 |
| | Arg$^{153}$ NH2 | 3.3 | | | |
| O1A | Gly$^{156}$ N | 2.8 | | | |
| | Gly$^{154}$ N | 3.3 | | | |

(v) CoA van der Waals interactions

| CoA Atom | Protein Residue/Ac-TRYP Atom | Distance (Å) | CoA Atom | Protein Residue Atom | Distance (Å) |
|---|---|---|---|---|---|
| S1P | Cys$^{181}$ CA | 3.6 | CDP | Leu$^{146}$ CG | 3.6 |
| | Ser$^{186}$ CB | 3.9 | | Leu$^{146}$ CD2 | 3.9 |
| | Ac-TRYP C11 | 3.4 | C5B | Val$^{189}$ CG1 | 3.7 |
| C2P | Ac-TRYP C11 | 3.5 | C2A | Arg$^{153}$ CB | 3.6 |
| C6P | Glu$^{47}$ CA | 3.8 | C6A | Arg$^{153}$ CB | 3.7 |
| C9P | Arg$^{153}$ CZ | 3.6 | C8A | Gly$^{154}$ CA | 3.7 |

Ac-TRYP

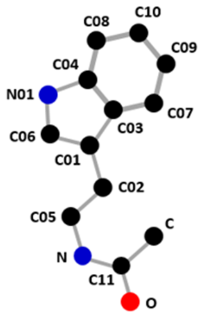


CoA

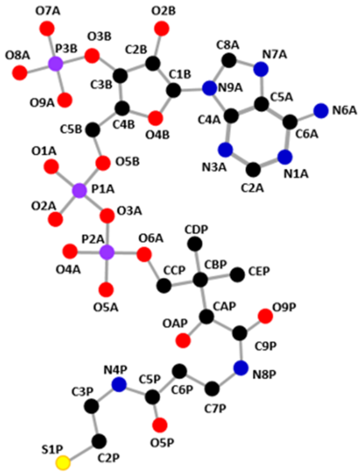

**Supplementary Table 2. Detailed protein-reactant contacts of Dat/Ac-CoA and Dat/CoA**

The number represents the distance (Å) between ligand atoms and protein residue atoms. Detail of ligands with labeled atoms are shown in the right panel.

| (i) Ac-CoA salt bridge | | | | | |
|---|---|---|---|---|---|
| CoA Atom | Protein Residue Atom | Distance (Å) | | | |
| O2A | Lys$^{192}$ NZ | 2.7 | | | |
| O8A | Lys$^{192}$ NZ | 2.7 | | | |
| (ii) Ac-CoA hydrogen bonds | | | | | |
| CoA Atom | Protein Residue Atom | Distance (Å) | CoA Atom | Protein Residue Atom | Distance (Å) |
| O | Leu$^{146}$ N | 3.0 | O1A | Gly$^{156}$ N | 2.8 |
| N4P | Leu$^{146}$ O | 2.9 | O4A | Ala$^{158}$ N | 2.9 |
| O9P | Val$^{148}$ N | 3.1 | O5A | Gly$^{154}$ N | 2.9 |
| | Arg$^{153}$ NH2 | 3.3 | | | |
| (iii) Ac-CoA van der Waals interactions | | | | | |
| CoA Atom | Protein Residue Atom | Distance (Å) | CoA Atom | Protein Residue Atom | Distance (Å) |
| CH3 | Val$^{179}$ CG1 | 3.8 | C2A | Arg$^{153}$ CB | 3.6 |
| C9P | Arg$^{153}$ CZ | 3.6 | | Arg$^{153}$ CD | 3.9 |
| CCP | Ala$^{158}$ CB | 3.8 | C5A | Gly$^{154}$ CA | 3.8 |
| CDP | Leu$^{146}$ CG | 3.6 | C6A | Arg$^{153}$ CB | 3.8 |
| | Val$^{148}$ CG2 | 3.9 | C8A | Gly$^{154}$ CA | 3.7 |
| C5B | Val$^{189}$ CG2 | 3.8 | | | |

Ac-CoA

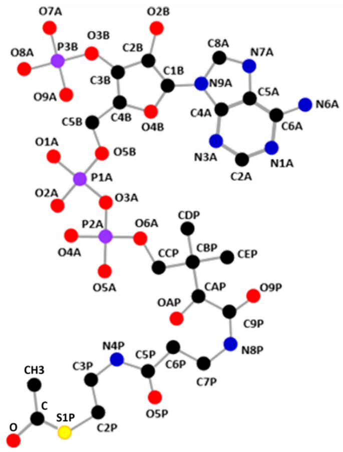

| (i) CoA salt bridge | | | | | |
|---|---|---|---|---|---|
| CoA Atom | Protein Residue Atom | Distance (Å) | | | |
| O2A | Lys$^{192}$ NZ | 2.7 | | | |
| O9A | Lys$^{192}$ NZ | 2.8 | | | |
| O7A | Arg$^{188}$ NH2 | 3.2 | | | |
| (ii) CoA hydrogen bonds | | | | | |
| CoA Atom | Protein Residue Atom | Distance (Å) | CoA Atom | Protein Residue Atom | Distance (Å) |
| S1P | Ser$^{182}$ N | 3.3 | O1A | Gly$^{154}$ N | 3.2 |
| | Ser$^{186}$ OG | 3.1 | | Gly$^{156}$ N | 3.0 |
| N4P | Leu$^{146}$ O | 2.8 | O4A | Ala$^{158}$ N | 2.9 |
| O9P | Val$^{148}$ N | 3.0 | O5A | Gly$^{154}$ N | 2.9 |
| | Arg$^{153}$ NH2 | 3.4 | O7A | Arg$^{188}$ NE | 2.7 |
| (iii) CoA van der Waals interactions | | | | | |
| CoA Atom | Protein Residue Atom | Distance (Å) | CoA Atom | Protein Residue Atom | Distance (Å) |
| S1P | Cys$^{181}$ CA | 3.7 | C1B | Try$^{185}$ CG | 3.9 |
| | Ser$^{186}$ CB | 3.9 | C2A | Arg$^{153}$ C | 3.9 |
| C3P | Ser$^{186}$ CB | 3.6 | | Gly$^{154}$ CA | 3.7 |
| C6P | Glu$^{47}$ CA | 3.8 | C6A | Arg$^{153}$ CB | 3.8 |
| C7P | Asp$^{46}$ CG | 3.8 | C8A | Try$^{185}$ CE2 | 3.6 |
| C9P | Arg$^{153}$ CZ | 3.7 | | Try$^{185}$ CZ | 3.8 |
| CAP | Arg$^{153}$ CD | 3.8 | | Try$^{185}$ CD2 | 3.6 |
| CCP | Ala$^{158}$ CB | 3.9 | | Try$^{185}$ CG | 3.7 |
| CDP | Leu$^{146}$ CG | 3.7 | | | |
| CEP | Ser$^{186}$ CA | 3.9 | | | |
| | Ser$^{186}$ CB | 3.9 | | | |

CoA

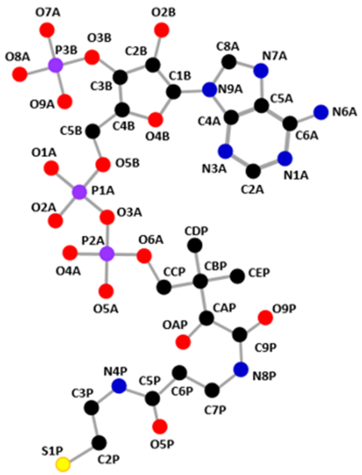

**Supplementary Table 3. The distances between the critical residues in different forms of Dat**

| Location | Critical residues /Atoms | Distance (Å) between two residues | | | |
|---|---|---|---|---|---|
| | | Dat | Dat/Ac-CoA | Dat/Ac-CoA/tryptophol | Dat/CoA/Ac-TRYP |
| Inter-helix | | | | | |
| α1-α5 | D46/OD2 & R153/NH2 | 5.0 | 2.7 | 2.7 | 2.7 |
| α9-α7 | H220/ND1 & S183/OG | 4.0 | 2.7 | 2.7 | 2.7 |
| α9-α7 | H220/O & H184/N | 6.8 | 2.9 | 2.9 | 2.9 |
| α9-α7 | H220/Cg & Y185/Cg | 4.6 | 4.9 | 4.9 | 4.9 |
| α1-α7 | E47/OE & S182/OG | 3.3 | 2.6 | 2.6 | 2.6 |
| α2-α4 | Y64/Cg & M121/SD | 7.5 | 5.3 | 5.3 | 5.3 |
| α4-α4 | F114/Cg & M121/SD | 9.4 | 7.0 | 7.0 | 7.0 |
| Note: Cg represents the centroid of the aromatic ring. | | | | | |
