# Supplementary material for: An essential role of acetyl coenzyme A in the catalytic cycle of insect arylalkylamine N-acetyltransferase
Source: Commun Biol. 2020 Aug 14;3:441. doi: 10.1038/s42003-020-01177-9 (PMC7427786; doi:10.1038/s42003-020-01177-9)
Supplement: Supplementary file 2 — Description of Additional Supplementary Files [file 42003_2020_1177_MOESM2_ESM.pdf]

Descriptions of Additional Supplementary Files

File name: Supplementary Data 1

Description: Data collection and refinement statistics as shown in Fig. 6a-c

File name: Supplementary Data 2

Description: Associated raw data in Fig. 6a-c: coordinates

File name: Supplementary Data 3

Description: Associated raw data in Fig. 6a-c: structure factors

File name: Supplementary Data 4

Description: Associated raw data in Fig. 6a-c: calculated mFo-DFc_map coordinating with the structures (pymol file)
