# Supplementary material for: An essential role of acetyl coenzyme A in the catalytic cycle of insect arylalkylamine N-acetyltransferase
Source: Commun Biol. 2020 Aug 14;3:441. doi: 10.1038/s42003-020-01177-9 (PMC7427786; doi:10.1038/s42003-020-01177-9)
Supplement: Supplementary file 3 — Supplementary Data 1 [file 42003_2020_1177_MOESM3_ESM.docx]

**Data collection and refinement statistics of product release from ternary structures as shown in Fig. 6a-c**

| Structure | Dat/CoA/Ac-PEA | Dat/CoA/Ac-PEA  + Ac-CoA | Dat/CoA/Ac-PEA  + water |
| --- | --- | --- | --- |
| Data collection | | | |
| Space group | P2_1_2_1_2_1_ | P2_1_2_1_2_1_ | P2_1_2_1_2_1_ |
| Cell dimensions | | | |
| a, b , c (Å) | 44.08, 56.65, 84.04 | 43.66, 56.29, 83.69 | 43.80, 56.59, 83.98 |
| α, β , γ (º) | 90, 90, 90 | 90, 90, 90 | 90, 90, 90 |
| Resolution (Å) | 30-1.34 (1.39-1.34) | 30-1.45 (1.50-1.45) | 30-1.57 (1.63-1.57) |
| R_p.i.m._ (%) | 1.9 (15.7) | 2.6 (29.1) | 1.6 (9.6) |
| I/σ | 35.5 (5.3) | 25.6 (2.4) | 40.8 (7.9) |
| Completeness (%) | 99.3 (94.4) | 98.9 (92.8) | 96.3 (93.7) |
| Redundancy | 6.0 (5.9) | 4.5 (3.3) | 3.7 (3.7) |
| Refinement | | | |
| Resolution (Å) | 25.11-1.34 (1.39- 1.34) | 28.15-1.45 (1.50-1.45) | 25.09-1.57 (1.63-1.57) |
| Number of reflections | 47573 (4616) | 36882 (3411) | 28662 (2720) |
| R_work_/R_free_ | 32.47/35.09 (33.44/36.45) | 27.12/30.91 (30.67/38.06) | 27.61/31.82 (25.16/33.38) |
| Number of atoms |  |  |  |
| Protein | 1688 | 1687 | 1688 |
| B-factor |  |  |  |
| Protein | 12.61 | 18.36 | 13.49 |
| RMSD |  |  |  |
| Bond length (Å) | 0.02 | 0.02 | 0.02 |
| Bond angle (°) | 2.10 | 2.00 | 2.03 |
| Ramachandran plot |  |  |  |
| Favored regions (%) | 97.12 | 97.6 | 97.12 |
| Allowed regions (%) | 2.88 | 2.40 | 2.88 |
| Outliers | 0.00 | 0.00 | 0.00 |
