# Supplementary material for: An essential role of acetyl coenzyme A in the catalytic cycle of insect arylalkylamine N-acetyltransferase
Source: Commun Biol. 2020 Aug 14;3:441. doi: 10.1038/s42003-020-01177-9 (PMC7427786; doi:10.1038/s42003-020-01177-9)
Supplement: Supplementary file 8 — Reporting Summary [file 42003_2020_1177_MOESM8_ESM.pdf]

nature research

Corresponding author(s): Ping-Chiang Lyu

Last updated by author(s): Jul 13, 2020

Reporting Summary

Nature Research wishes to improve the reproducibility of the work that we publish. This form provides structure for consistency and transparency in reporting. For further information on Nature Research policies, see Authors & Referees and the Editorial Policy Checklist.

Statistics

For all statistical analyses, confirm that the following items are present in the figure legend, table legend, main text, or Methods section.

n/a	Confirmed	
☐	☒	The exact sample size (*n*) for each experimental group/condition, given as a discrete number and unit of measurement
☐	☒	A statement on whether measurements were taken from distinct samples or whether the same sample was measured repeatedly
☒	☐	The statistical test(s) used AND whether they are one- or two-sided<br>*Only common tests should be described solely by name; describe more complex techniques in the Methods section.*
☒	☐	A description of all covariates tested
☒	☐	A description of any assumptions or corrections, such as tests of normality and adjustment for multiple comparisons
☐	☒	A full description of the statistical parameters including central tendency (e.g. means) or other basic estimates (e.g. regression coefficient) AND variation (e.g. standard deviation) or associated estimates of uncertainty (e.g. confidence intervals)
☒	☐	For null hypothesis testing, the test statistic (e.g. *F*, *t*, *r*) with confidence intervals, effect sizes, degrees of freedom and *P* value noted<br>*Give P values as exact values whenever suitable.*
☒	☐	For Bayesian analysis, information on the choice of priors and Markov chain Monte Carlo settings
☒	☐	For hierarchical and complex designs, identification of the appropriate level for tests and full reporting of outcomes
☒	☐	Estimates of effect sizes (e.g. Cohen's *d*, Pearson's *r*), indicating how they were calculated

Our web collection on statistics for biologists contains articles on many of the points above.

Software and code

Policy information about availability of computer code

Data collection	The diffraction data were processed with HKL2000. Data collection statistics are shown in Table 1.
Data analysis	All the structures were determined using molecular replacement with Phaser-MR (simple interface) of PHENIX using a previously reported Dat/Ac-CoA structure (PDB code: 3TE4) as the search model. Multiple rounds of refinement were carried out using WinCoot and PHENIX. Molecular graphics of structures and electron density maps were visualized using PyMol Molecular Graphics System (Ver. 2.3 Schrödinger, LLC). Ramachandran plot statistics of the final models were calculated by PROCHECK. Refinement statistics are shown in Table 1.

For manuscripts utilizing custom algorithms or software that are central to the research but not yet described in published literature, software must be made available to editors/reviewers. We strongly encourage code deposition in a community repository (e.g. GitHub). See the Nature Research guidelines for submitting code & software for further information.

Data

Policy information about availability of data

All manuscripts must include a data availability statement. This statement should provide the following information, where applicable:

- Accession codes, unique identifiers, or web links for publicly available datasets
- A list of figures that have associated raw data
- A description of any restrictions on data availability

-All relevant data are available upon reasonable request.
-Coordinates and structure factors have been deposited in the protein data bank, under accession codes PDB ID 6K80 (Dat/AcCoA/Tryptophol complex), 5GI9 (Dat/CoA/Ac-TRYP complex) and 5GI5 (Dat/CoA complex).
-Source data for Fig. 6a-c were provided in Supplementary Data 1-4.

Field-specific reporting

Please select the one below that is the best fit for your research. If you are not sure, read the appropriate sections before making your selection.

☒ Life sciences ☐ Behavioural & social sciences ☐ Ecological, evolutionary & environmental sciences

For a reference copy of the document with all sections, see nature.com/documents/nr-reporting-summary-flat.pdf

Life sciences study design

All studies must disclose on these points even when the disclosure is negative.

Sample size	n/a,the sample size was chosen based on common practice in the field.
Data exclusions	No data were excluded.
Replication	All the experiments in the manuscript were successfully reproduced
Randomization	n/a
Blinding	n/a

Reporting for specific materials, systems and methods

We require information from authors about some types of materials, experimental systems and methods used in many studies. Here, indicate whether each material, system or method listed is relevant to your study. If you are not sure if a list item applies to your research, read the appropriate section before selecting a response.

Materials & experimental systems

n/a	Involved in the study	
☒	☐	Antibodies
☒	☐	Eukaryotic cell lines
☒	☐	Palaeontology
☒	☐	Animals and other organisms
☒	☐	Human research participants
☒	☐	Clinical data

Methods

n/a	Involved in the study	
☒	☐	ChIP-seq
☒	☐	Flow cytometry
☒	☐	MRI-based neuroimaging
